# Low-Cost Biomass Nanofibers from Chitosan and Phytic Acid for Efficient Uranium Extraction

**DOI:** 10.3390/polym17202725

**Published:** 2025-10-10

**Authors:** Zixu Ren, Dongqi Geng, Dingyang Chen, Minsi Shi, Qing Bai, Rui Zhao

**Affiliations:** 1 Key Laboratory of Polyoxometalate and Reticular Material Chemistry of Ministry of Education, Faculty of Chemistry, Northeast Normal University, Changchun 130024, China; zixuren@nenu.edu.cn (Z.R.); gengdongqi613@nenu.edu.cn (D.G.); chendy799@nenu.com (D.C.); shiminsi@126.com (M.S.); 2 College of New Materials and New Energies, Shenzhen Technology University, Shenzhen 518118, China

**Keywords:** uranium extraction, electrospinning, biomass nanofiber, chitosan, phytic acid

## Abstract

Exploring materials for the uranium extraction from seawater holds great significance for the sustainable development of the nuclear industry. Though many adsorbents have been investigated to extract uranium, they still suffer from the issues of low adsorption performance and high production cost. In this work, biomass nanofiber adsorbents (PA-CS NFs) were prepared by the electrospinning of chitosan followed by functionalization with phytic acid. Based on the cost analysis, the preparation expense of PA-CS NFs was $16.4 kg^−1^, lower than those of common synthetic polymer adsorbents. In addition, PA-CS NFs showed fast removal kinetics (equilibrium time = 60 min), high uptake capacity (457.8 mg g^−1^), and good selectivity (the ratio of uranium/competing ion capacities > 3.8) from uranium spiked solution. PA-CS NFs also exhibited the ability to remove trace uranyl ions (distribution coefficient = 4.7 × 10^5^ mL g^−1^) and satisfy recycling capacity. The experimental tests and theoretical calculations confirmed that the phosphate groups in the functionalized phytic acid displayed the main contribution to the uranyl ion adsorption, which had higher binding energy than the functional groups in chitosan. Benefiting from the good adsorption ability, low cost, and macroscopical membrane form, PA-CS NFs were applied to natural seawater for uranium extraction, and an extraction capacity of 4.52 mg g^−1^ could be achieved after 35 days’ testing. On account of the obtained results, this study offers an efficient and low-cost nanofiber adsorbent for uranium extraction.

## 1. Introduction

With the continuous demand of global energy and the increasingly serious environmental problems, developing nuclear energy has become a significant approach to alleviate the energy crisis and reduce greenhouse gas emissions [1]. Uranium is one of the key raw materials in nuclear reactions and plays an indispensable role in the nuclear energy industry [2]. However, it is reported that terrestrial uranium resources are limited and will be exhausted in approximately one hundred years [3]. In contrast, the ocean contains approximately 4.5 billion tons of uranium reserves, and the nuclear energy industry will have an inexhaustible supply of raw materials if these resources are fully exploited [4]. In other words, efficient uranium extraction from seawater (UES) has significant strategic and research value. Nevertheless, UES still faces severe challenges, such as extremely low uranium concentration in seawater (3.3 μg L^−1^) and the presence of large amounts of competitive ions [5]. Furthermore, the presence of marine organisms and bacteria leads to biofouling, which impairs long-term performance [6]. As a result, exploring effective methods and materials for the UES is a research hotspot, which provides a lot of room for further research.

Up to now, many technologies, including ion exchange, biotransformation, co-precipitation, photocatalysis, electrochemical method, adsorption, etc., have been investigated for uranium extraction [4,7,8,9,10]. However, traditional ion exchange, biotransformation, and co-precipitation suffer from low performance. Emerging photocatalytic and electrochemical methods require external assistance and consume additional energy. Among them, adsorption seems to be the most promising method for large-scale uranium extraction from seawater, owing to its simplicity, easy operation, high efficiency, and no need for external field assistance [11]. To achieve efficient UES, different adsorbents have been investigated, including inorganic metal compounds, carbon materials, synthetic polymers, natural polymers, and advanced porous materials [12,13,14,15,16]. For the practical extraction in open seas, the adsorbents should have features of good adsorption performance, low production cost, and macroscopic engineered morphology. However, most uranium adsorbents fall short in at least one of these characteristics, limiting their effectiveness for real applications. This situation drives the development of other materials that can more effectively achieve practical uranium extraction applications.

Biomass has the major property of taking materials from nature, and is a potential candidate to obtain a low-cost adsorbent [17]. In our previous studies, some low-cost uranium adsorbents have been prepared from biomasses [18,19]. However, the as-obtained materials are powders that are not conducive to usage in marine environments. Electrospinning is a feasible technology to produce fibers with diameters in nanometer scale. The electrospun nanofibers show high porosity, easy functionalization, and macroscopic membrane form, making them a hot topic as adsorbents [20,21,22,23]. To obtain the electrospun nanofibers, a polymer solution should be prepared to conduct the spinning process. Chitosan is a natural linear polysaccharide and is the second most abundant natural polymer after cellulose. On its polymer chain, there are a lot of amino groups and hydroxyl groups that could be applied to adsorb many ions and molecules [24,25]. In addition, chitosan inherently exhibits antibacterial properties, which help maintain high adsorption efficiency in seawater by inhibiting the formation of biofouling [26]. Though some electrospun chitosan-based nanofibers have been studied for uranium adsorption, synthetic polymers usually exist in the final prepared fibers [27,28]. However, our objective is to develop pure biomass-based nanofibers with fully biodegradable and cost-effective properties. Previous studies have shown that pure chitosan displayed unsatisfactory adsorption ability toward uranyl ions (UO_2_^2+^). The functionalization of chitosan nanofibers by other biomasses is needed. Phytic acid (PA) is a natural polyphosphoric compound, and one PA molecule has six phosphate groups. Phosphate groups are reported to be effective chelators to bind UO_2_^2+^ ions, owing to their electron-rich ability [29]. Based on this property, PA has been widely studied to modify different substrate materials for UO_2_^2+^ adsorption [30,31]. Thus, the composition of chitosan nanofiber with phytic acid will produce novel adsorbents for uranium extraction.

In this work, pure chitosan nanofibers were prepared from the electrospinning of chitosan/polyethylene oxide (PEO) mixture solution followed by PEO removal. Then, PA was modified onto chitosan nanofibers via the non-covalent assembly to obtain pure biomass-based nanofibers. The physical and chemical structures of the prepared biomass-based nanofibers were characterized, and their uranium adsorption performance was well studied. The biomass-based nanofibers have the following advantages: (i) the preparation process is easy to operate and cost-effective; (ii) pure biomass-based nanofibers are green and biodegradable, which would not lead to secondary pollution; (iii) electrospun nanofibers show macroscopic membrane morphology that meets the requirements of practical applications; and (iv) PA modification could greatly improve uranium adsorption performance. As a result, the biomass-based nanofibers exhibited fast and high uptake, satisfactory selectivity, and good regenerability. Moreover, an extraction capacity of 4.52 mg g^−1^ could be achieved from natural seawater by the nanofiber adsorbent after 35 days’ test. Overall, this work provides a novel biomass-based adsorbent and demonstrates its application for uranium extraction from seawater.

## 2. Materials and Methods

### 2.1. Materials and Instruments

Chitosan (degree of deacetylation > 95%, viscosity > 400 mPa·s) and phytic acid (70% in H_2_O) were obtained from Aladdin Biochemical Technology Co., Ltd. (Shanghai, China). Poly(ethylene oxide) (PEO, Mv = 300,000 Da) and UO_2_(NO_3_)_2_·6H_2_O were purchased from Macklin Inc. (Shanghai, China). Acetic acid (99.5%) and other inorganic salts were obtained from Energy Chemical (Shanghai, China). All reagents were used without further purification. Field emission scanning electron microscopy (SEM, Shimadzu SSX-550, Shimadzu Corporation, Kyoto, Japan) and transmission electron microscopy (TEM, Hitachi S570, Hitachi High-Tech Corporation, Tokyo, Japan) with energy-dispersive X-ray spectroscopy (EDS) were applied to observe morphology. Fourier-transform infrared (FT-IR) spectra were conducted on a Nicolet iS50 Fourier transform infrared spectrometer (Thermo Fisher Scientific, Waltham, MA, USA). Thermogravimetric analysis (TGA) curves were recorded on a Mettler Toledo thermal analyzer (METTLER TOLEDO, Zurich, Switzerland) under an air atmosphere. An X-ray photoelectron spectroscopy (XPS) test was carried out on the Thermo ScientificTM NexsaTM XPS system (Thermo Fisher Scientific, Waltham, MA, USA).

### 2.2. Fabrication of Pure Chitosan Nanofibers

Chitosan powders were first dissolved in glacial acetic acid (90%) to obtain chitosan solution (4 wt%). PEO powders were dissolved in deionized water to obtain PEO solution (4 wt%). These two solutions were mixed with the chitosan and PEO mass ration of 3.5:1.0. The mixed solution was added into the syringe (10 mL), and the aluminum foil acted as the collector. A voltage of 18 kV was applied between the syringe and collector to conduct the electrospinning. A feed rate of 1 min h^−1^ was applied to force the spinning solution through the spinneret during the electrospinning process. After electrospinning for sufficient time, the nanofibers could be collected from the aluminum foil. Then, the as-prepared nanofibers were immersed in NaOH solution (1 M) for 4 h. The treated nanofibers were fully washed with deionized water and dried in a vacuum oven for 24 h to produce pure chitosan nanofibers.

### 2.3. Modification of Phytic Acid on Chitosan Nanofibers

Dried chitosan nanofibers (100 mg) were immersed in 20 mL phytic acid solution (10 wt%) for 24 h. The reacted nanofibers were washed with deionized water and dried in a vacuum oven for 24 h to obtain phytic acid modified chitosan nanofibers. For comparison, the concentration of phytic acid solution was also adjusted to 1 wt%, 5 wt%, 20 wt%, and 30 wt%, respectively.

### 2.4. Adsorption Experiments from Uranium Spiked Sotluion

Uranium spiked solution was prepared by adding UO_2_(NO_3_)_2_·6H_2_O in deionized water. The concentration of uranium in the solution was measured by a UV–visible spectrophotometer using Arsenazo III assay and an inductively coupled plasma mass spectrometer (ICP-MS). All adsorption experiments were performed in triplicate to ensure reproducible results. The adsorption capacity (*q*) toward uranium was calculated using the following equation:

(1) $$q\;(mg/g)=\frac{\left(C_{0}-C_{e}\right)V}{W}$$

where *C*_0_ and *C_e_* are the initial concentration and equilibrium concentration of uranium in the test solution (mg L^−1^), V is the volume of the test solution (L), and *W* is the mass of the adsorbent (*g*).

To investigate the effect of initial pH on the adsorption performance of the adsorbent for U(VI), adsorption experiments were conducted using 20 mL of uranyl solution with a concentration of 50 mg L^−1^. The initial pH of the solution was adjusted to a range of 4–9 using 0.01 mol L^−1^ HCl or NaOH. Subsequently, 6 mg of PA-CS NFs were added to the solution, which was stirred until the adsorption equilibrium. Then, the concentration of uranium in the solution was measured and the adsorption capacity was calculated.

Adsorption kinetic tests were performed by introducing 24 mg of adsorbent into 80 mL of uranyl solution (initial concentration: 50 mg L^−1^). Under continuous stirring, samples were collected at specific time points for subsequent determination of the adsorption capacity.

Adsorption isotherm measurements were conducted through a series of uranyl solutions with initial concentrations ranging from 5 to 250 mg L^−1^, and 3 mg of adsorbent was added to each 30 mL of solution. The mixtures were stirred until the adsorption equilibrium. Then, the equilibrium concentration was measured, and the adsorption capacity was determined. The isotherm data were analyzed using both Langmuir and Freundlich isotherm models, whose linearized equations are expressed as follows:

Langmuir isotherm (homogeneous and monolayer adsorption):

(2) $$\frac{C_{e}}{q_{e}}=\frac{C_{e}}{q_{m}}+\frac{1}{{bq}_{m}}$$

Freundlich isotherm (heterogeneous and multilayer adsorption):

(3) $$logq_{e}=logK_{F}+\frac{1}{n}logC_{e}$$

where *q_e_* is the equilibrium adsorption capacity (mg g^−1^), *C_e_* is the equilibrium concentration (mg L^−1^), and *q_m_* and b are Langmuir constants related to maximum adsorption capacity and binding energy, respectively; *K_F_* and n are empirical constants that indicate the Freundlich constant and heterogeneity factor, respectively.

Trace adsorption experiments were conducted by adding 20 mg of adsorbent into 20 mL of uranyl solution with an initial concentration of 1 mg L^−1^. At predetermined time intervals, 2 mL of the solution were withdrawn for subsequent concentration analysis. The distribution coefficient (*K_d_*) was calculated using the following formula:

(4) $$K_{d}=\left(\frac{C_{0}-C_{e}}{C_{e}}\right)\times \frac{V}{m}$$

Adsorption selectivity under the coexistence of multiple competitive ions was evaluated. A solution containing various ions (U(VI), V(V), Sr(II), Cu(II), Co(II), Zn(II), Ni(II), Na(I), Mg(II), Ca(II), K(I), Fe(III), and Ba(II)) was prepared. The concentration of each ion was fixed at 10 mg L^−1^. The adsorbent (2 mg) was added to the mixed metal–ion solution (20 mL). After the adsorption equilibrium, the concentration of each ion was measured, and their adsorption capacities were calculated.

The regeneration capability of the adsorbent was evaluated through ten consecutive adsorption–desorption cycles. In each cycle, 6 mg of PA-CS NFs were exposed to 20 mL of uranyl solution (50 mg·L^−1^) until saturation was achieved. The uranium-loaded adsorbent was then treated with an eluent (100 mmol L^−1^ ethylenediaminetetraacetic acid (EDTA), 30 mL) for regeneration purposes. Subsequently, the adsorbent was collected and thoroughly washed with water until a neutral pH was achieved. The regenerated adsorbent was then reused in the next adsorption experiment.

### 2.5. Uranium Extraction from Real Seawater

Natural seawater was collected from Bohai Sea near the east coast of Qingdao city, China. The seawater was filtrated by a 0.22 µm membrane filter to remove marine biological entities and suspended particulate contaminants. An amount of 100 L of filtrated seawater was forced to pass through the nanofiber adsorbents (5 mg) to conduct the extraction experiment. During the extraction process, 10 mL aliquots of seawater were collected at specific time intervals (5, 10, 15, 20, 25, 30, and 35 days) to determine the extraction capacity.

## 3. Results

### 3.1. Synthesis of Nanofiber Adsorbent

**Figure 1:**
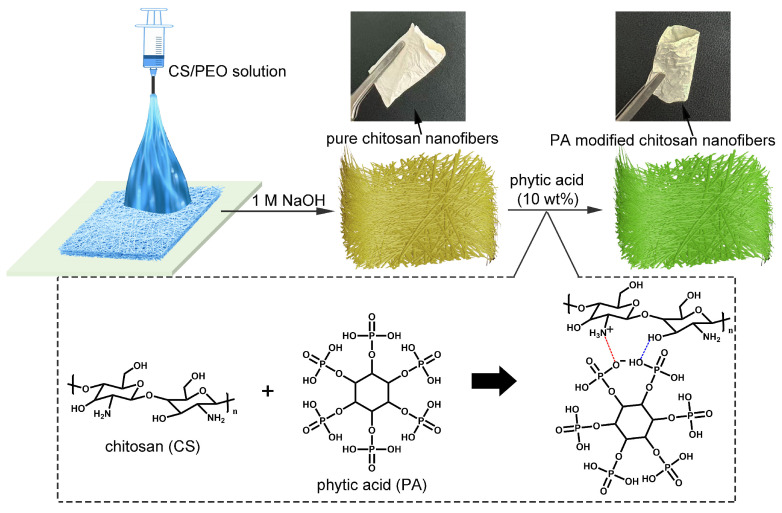
Preparation process diagram of PA-CS NFs.

The schematic synthesis route of the biomass-based nanofiber adsorbent is shown in Figure 1. In this work, PEO was applied as the electrospinning assistant to promote the formation of chitosan nanofibers. After the removal of the PEO template by the eluent, pure chitosan nanofibers (CS NFs) were prepared. This process was also adopted by other studies [32,33]. Although the synthetic polymer (PEO) was employed to facilitate the electrospinning of chitosan, the PEO present in the eluent could be readily recycled through the neutralization of NaOH and subsequent water evaporation. Consequently, the recycled PEO could be reused for the next preparation cycle of CS NFs. Due to the sustainability of PEO, we think the prepared adsorbents are based on the biomasses. For the functionalization of phytic acid (PA), CS NFs were gently immersed in PA solution. During this process, the PA molecules were modified on CS NFs via the multiple interactions of electrostatic attraction and hydrogen bonds. Notably, after the removal of the functionalized nanofibers, the PA solution could be recycled for the next functionalization cycle via replenishing PA to reduce the generation of waste liquid. After the functionalization, the PA modified chitosan nanofibers (PA-CS NFs) still maintained the good flexibility of electrospun nanofibers. The mechanical properties of PA-CS NFs were studied by stress–strain curves (Figure S1). The tensile strength of PA-CS NFs was found to be 1.1 ± 0.3 MPa, and the elongation at break was 2.8 ± 0.2%. The obtained mechanical properties could ensure the PA-CS NFs’ usage as adsorbents in static adsorption and dynamic column adsorption. Moreover, the preparation cost of PA-CS NFs was evaluated, which was calculated to be $16.40 kg^−1^ (Table 1). This cost was lower than the common synthetic polymer adsorbents [34,35].

### 3.2. Morphological Analysis

**Figure 2:**
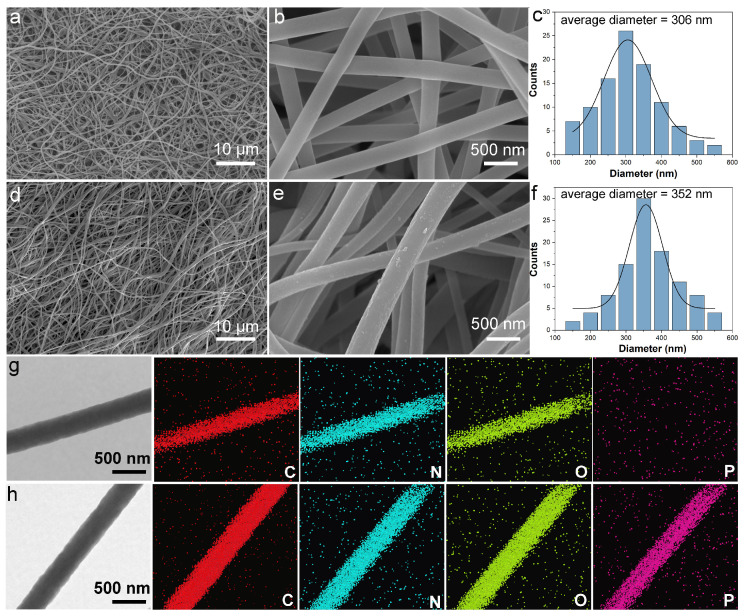
SEM images and diameter distribution curves of CS NFs (**a**–**c**) and PA-CS NFs (**d**–**f**). TEM images and corresponding EDS elemental mappings of CS NFs (**g**) and PA-CS NFs (**h**).

CS NFs showed the typical morphology of electrospun fibers with unordered and non-woven fibrous structures (Figure 2a). In the magnified SEM images, the surfaces of CS NFs were smooth (Figure 2b). However, the surfaces of PA-CS NFs became coarser (Figure 2d,e) and the average diameter increased to 352 nm from 306 nm (Figure 2c,f) owing to the swelling and the action of PA. Compared with solid materials, the abundant porosity among the electrospun nanofibers could expose more active sites and accelerate the diffusion of targeted ions. In the TEM images (Figure 2g,h), both CS NFs and PA-CS NFs were of a homogeneous phase without a core–shell structure, suggesting that the interaction between PA and CS NFs was the chemical binding rather than the physical coating. As shown in the related EDS mapping images, obvious P element could be observed in PA-CS NFs in comparison with CS NFs, confirming the PA modification.

### 3.3. Characterization of Chemical Structures

**Figure 3:**
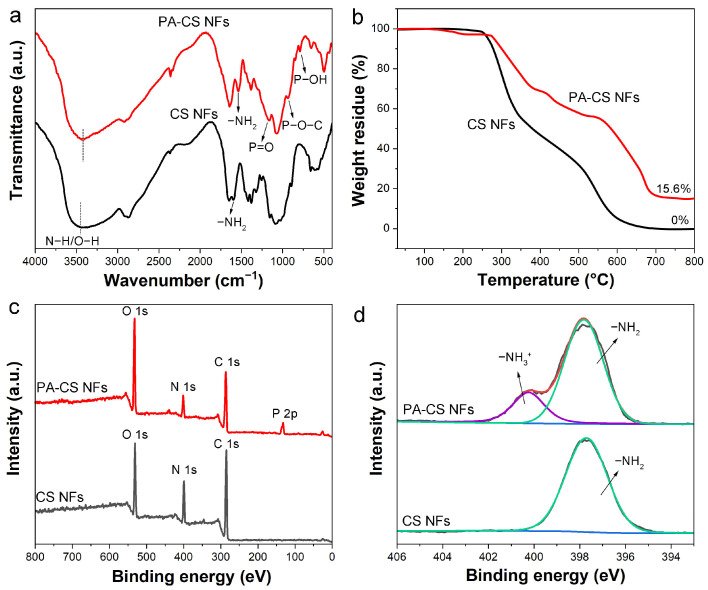
(**a**) FT-IR spectra, (**b**) TGA curves, (**c**) XPS survey spectra, and (**d**) high-resolution N 1s spectra of CS NFs and PA-CS NFs.

The chemical components of the nanofibers were further studied by FT-IR and XPS characterizations. A comparison of the FT-IR spectra of PA-CS NFs and CS NFs revealed the presence of characteristic peaks related to PA in the spectrum of PA-CS NFs, confirming the successful incorporation of PA into the CS nanofibers (Figure 3a). The spectrum of CS NFs showed the peak at 1594 cm^−1^ belonging to the stretching vibration of –NH_2_ [24]. However, this peak shifted to 1536 cm^−1^ in the spectrum of PA-CS NFs. This shift was attributed to the protonation of amino groups that interacted with the phosphate groups in PA [18]. Moreover, the characteristic peak corresponding to N–H/O–H also detected a wavenumber change after the PA modification, which was due to the H-bond interaction [36]. The peaks in PA-CS NFs at 1161 cm^−1^, 934 cm^−1^, and 791 cm^−1^ were assigned to P=O, P–O–C, and P–OH, respectively [37], indicating the presence of PA. The analysis of FT-IR results verifies the composition between PA and CS NFs. We also conducted TGA experiments on PA-CS NFs and CS NFs to investigate the thermal stability of the samples and the PA loading (Figure 3b). The results revealed three distinct weight loss stages consistent with the molecule degradation process: evaporation of guest molecules and solvents, thermal decomposition of functional groups in the polymer, and degradation of the polymer backbone. CS NFs could be degraded completely at the high temperature of 800 °C. However, the PA-CS NFs finally showed the weight residue of 15.6% because of the remaining phosphorus compounds. Based on the previous report, the residual mass of PA in the TGA test was 30.1% [38]. Thus, the PA loading in PA-CS NFs was 51.8 wt% (15.6/30.1).

To further confirm the elemental composition of PA-CS NFs and CS NFs, X-ray photoelectron spectroscopy (XPS) analysis was conducted. As shown in Figure 3c, besides the elements N, O, and C, the XPS spectrum of PA-CS NFs exhibited the presence of P element due to the successful introduction of PA, which is consistent with the aforementioned results. Moreover, the high-resolution N 1s spectra were also analyzed (Figure 3d). The CS NFs exhibited only one peak at 397.7 eV, which was attributed to the –NH_2_. After the PA modification, a new peak appeared at 400.3 eV, corresponding to the protonated nitrogen –NH_3_^+^) from the electrostatic interaction between PA and CS [18]. The XPS results were consistent with the FT-IR findings, further confirming the successful synthesis of our target nanofibers. To verify the feasibility of using PA-CS NFs in seawater, their stability was evaluated in real seawater with high ionic strength and different pH values. As presented in Figure S2, the weight loss of PA-CS NFs remained extremely low across the pH range of 5.0~9.0. Notably, the pH of natural seawater is approximately 8.1, a value well within the aforementioned pH range where PA-CS NFs exhibited high stability. Therefore, PA-CS NFs possess the necessary stability for practical application in real marine environments.

### 3.4. Effect of Solution pH on Uranium Adsorption

**Figure 4:**
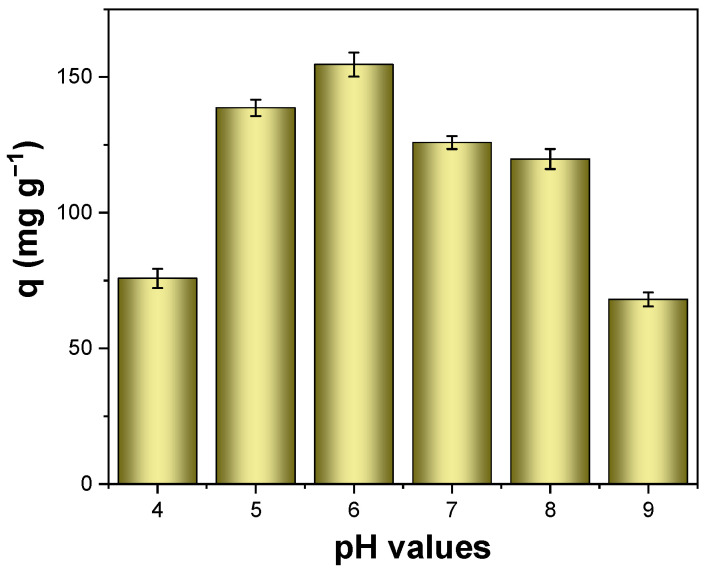
Adsorption capacities of PA-CS NFs toward uranium at different initial solution pH (C_0_ = 50 mg L^−1^, m/V = 0.3 g L^−1^).

The uranium adsorption performance of PA-CS NFs was first investigated via changing the initial pH value of uranium-spiked solution, and the adsorption experiments were conducted within a pH range from 4 to 9. Generally, the solution pH significantly influences the adsorption behavior of adsorbents, making it essential to determine the optimal pH value. As shown in Figure 4, it could be observed that the uranium adsorption capacity of PA-CS NFs was pH-dependent. The adsorption capacity gradually increased as the pH rose from 4 to 6 but declined slightly under weakly alkaline conditions. Under acidic conditions, uranium exists primarily as positively charged uranium ions. The existing H^+^ ions could compete with the uranyl ions. As the pH gradually increased, the concentration of H^+^ ions decreased, which weakened this competition, thereby enhancing the adsorption capacity. However, when the pH exceeded 6, the number of negatively charged uranium species (UO_2_(OH)_3_^−^, (UO_2_)_3_(OH)_7_^−^, etc.) began to increase [34]. Moreover, the functional groups –PO_4_H_2_, –NH_2_, –OH) in phytic acid and chitosan became progressively more negatively charged [39,40]. Thus, the electrostatic repulsion weakened the uranium uptake. Consequently, the optimal pH for uranium adsorption by PA-CS NFs was 6.

### 3.5. Effect of PA Modification

**Figure 5:**
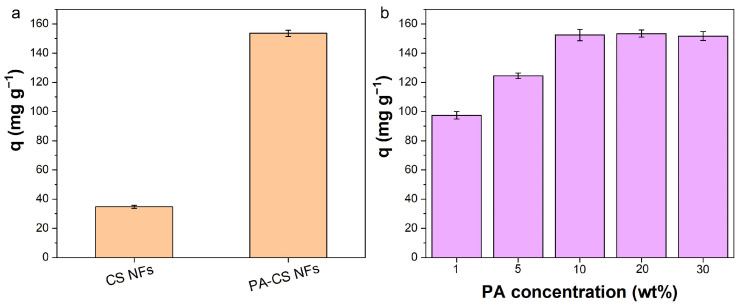
(**a**) Adsorption capacity comparison of PA-CS NFs and CS NFs. (**b**) Effect of PA solution concentration on the adsorption capacity of PA-CS NFs. (C_0_ = 50 mg L^−1^, m/V = 0.3 g L^−1^).

We compared the adsorption capacities of the obtained nanofibers (CS NFs and PA-CS NFs). As shown in Figure 5a, the adsorption capacity of PA-CS NFs was obviously higher than that of CS NFs. The result indicated that phosphate groups in PA were more effective than amino groups and hydroxyl groups in CS for binding uranyl ions. The introduction of PA could increase the affinity toward uranium, conforming to the design concept. Additionally, the loading of PA was also optimized via adjusting the concentration of PA solution when modifying the CS NFs (Figure 5b). Low PA concentration resulted in low PA loading on PA-CS NFs, and the uranium adsorption amount was not high. In this work, the used concentration of PA solution was 10 wt% for PA modification. However, if the concentration of PA solution further increased, the adsorption amounts no longer continued to increase because the site for PA loading in CS NFs had been fully occupied.

### 3.6. Adsorption Kinetics and Isotherm

**Figure 6:**
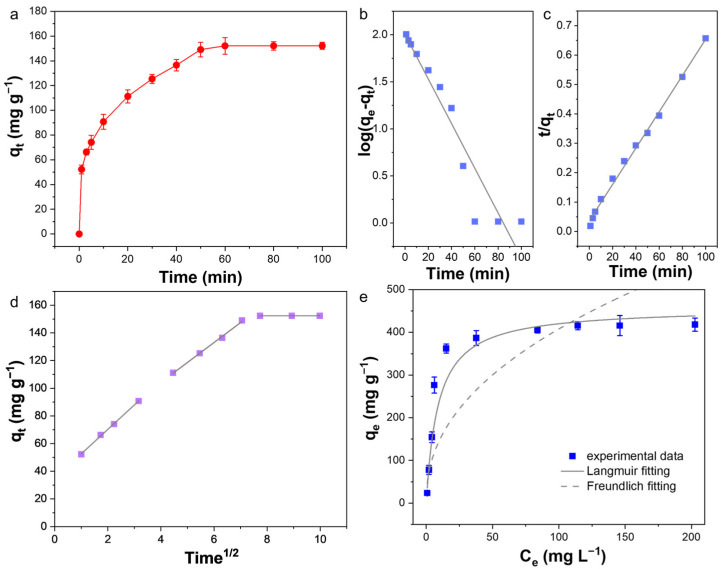
(**a**) Uranium adsorption kinetic curve (C_0_ = 50 mg L^−1^, m/V = 0.3 g L^−1^) and its related linear fitting of (**b**) the pseudo-first-order kinetic model, (**c**) the pseudo-second-order kinetic model, and (**d**) the Weber and Morris model. (**e**) Adsorption isotherm curve (C_0_ = 5~250 mg L^−1^, m/V = 0.1 g L^−1^).

A detailed investigation of the adsorption kinetics and adsorption isotherm of PA-CS NFs was conducted to gain an in-depth understanding of the adsorption behavior. To explore the adsorption rate, an adsorption kinetic study was carried out. As displayed in Figure 6a, the adsorption process consisted of two stages for uranium adsorption by PA-CS NFs. The adsorption rate increased rapidly within the first 20 min due to the abundance of binding sites and the high uranium concentration in the solution. Then, the adsorption rate gradually slowed down, and the adsorption process eventually reached equilibrium at 60 min. The fast adsorption kinetics were attributed to the high porosity of electrospun nanofibers. The pseudo-first-order kinetic model, pseudo-second-order kinetic model, and Weber and Morris model were further used to analyze the kinetic data. Based on the linear fitting curves (Figure 6b,c) and the fitted parameters (Table S1), the pseudo-second-order kinetic model could better describe the uranium adsorption by PA-CS NFs, suggesting the chemisorption process through the coordination. Furthermore, the fitting of the Weber and Morris model showed three stages (Figure 6d), corresponding to film diffusion, intra-particle diffusion, and the final equilibrium step, respectively. None of the fitting curves passed through the origin, indicating that the adsorption process was controlled by a multistep mechanism of both film diffusion and intra-particle diffusion [41].

To further evaluate the adsorption performance, isothermal adsorption experiments were performed within an initial U(VI) concentration range of 5~250 mg L^−1^. The results in Figure 6e showed that the U(VI) uptake by PA-CS NFs increased by increasing the initial concentration until reaching a plateau. These results confirm the strong affinity of PA-CS NFs toward uranyl ions. Furthermore, the Langmuir and Freundlich isothermal models were used to assess the adsorption equilibrium performance. The fitting results indicated that the Langmuir isotherm model aligned better with the adsorption equilibrium data than the Freundlich isotherm model (Table 2). Based on this model, the maximum adsorption capacity was calculated to be 457.8 mg g^−1^. We also compared the adsorption equilibrium time and adsorption capacity with other biomass-based adsorbents, and the adsorption performance of PA-CS NFs was higher than most of reported biomass-based adsorbents (Table 3).

### 3.7. Deep Removal, Selectivity, and Recyclability

**Figure 7:**
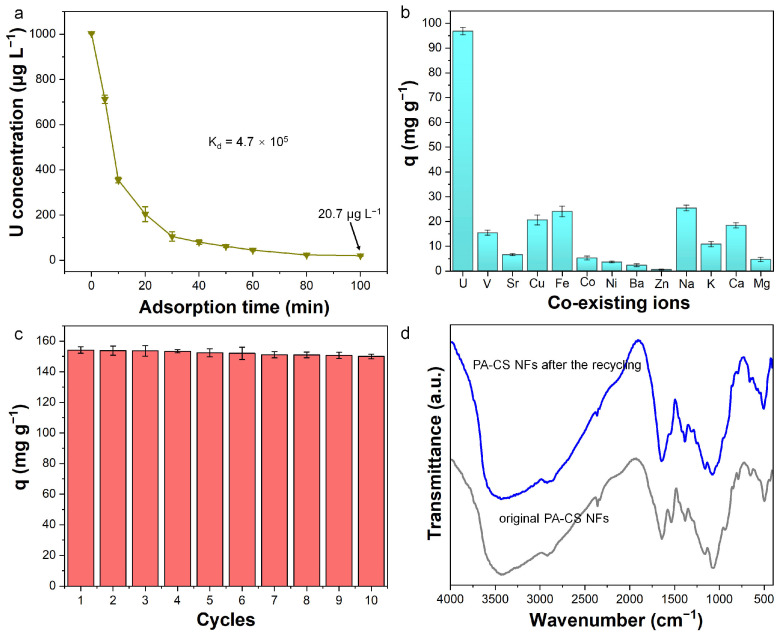
(**a**) The adsorption ability of PA-CS NFs toward trace uranium (C_0_ = 1 mg L^−1^, m/V = 1.0 g L^−1^). (**b**) Adsorption selectivity toward uranium in the coexistence of various metal ions (C_ion_ = 10 mg L^−1^, m/V = 0.1 g L^−1^). (**c**) Uranium adsorption capacity in ten successive adsorption–desorption cycles (C_0_ = 50 mg L^−1^, m/V = 0.3 g L^−1^). (**d**) FT-IR spectra of PA-CS NFs before and after ten adsorption–desorption cycles.

In real seawater, the uranium concentration is extremely low. Therefore, we evaluated the adsorption performance of PA-CS NFs toward trace uranium (Figure 7a). The concentration of uranium showed a significant decrease upon treatment with PA-CS NFs. The uranium concentration reduced from the initial 1 mg L^−1^ to 20.7 μg L^−1^ within 100 min below the drinking water standard (30 μg L^−1^) of the Environmental Protection Agency (EPA). Moreover, the distribution coefficient (K_d_) was calculated to be 4.7 × 10^5^ mL g^−1^. The results demonstrated the strong affinity of PA-CS NFs toward uranyl ions [16] and their ability to remove trace uranium.

To quantify the selective enrichment capability of PA-CS NFs for U(VI), 13 metal ions (U(VI), V(V), Sr(II), Cu(II), Co(II), Zn(II), Ni(II), Na(I), Mg(II), Ca(II), K(I), Fe(III), and Ba(II)) were dissolved together. The quantitative analysis of adsorption amount results is shown in Figure 7b. Even in the presence of multiple interfering metal ions, PA-CS NFs still exhibited high selectivity toward U(VI). For the competing ions, the adsorption toward Cu(II) and Fe(III) was high, mainly arising from the relatively strong coordination ability between the functional groups of PA-CS NFs and Cu(II)/Fe(III) ions. Notably, the uptake of U(VI) by PA-CS NFs remained more than 3.8 times higher than that of these two competing ions, demonstrating the material’s robust selectivity for U(VI) binding. With respect to real seawater systems, a small amount of these ions is likely to be co-adsorbed onto PA-CS NFs during uranium extraction.

The recyclability of PA-CS NFs was investigated through multiple adsorption–desorption cycles. In this work, 100 mmol L^−1^ EDTA solution was applied as the eluent. After ten adsorption–desorption cycles, the adsorption capacity could still maintain 97.3% of the value of the first cycle (Figure 7c). Moreover, the continuously increasing uranium concentration in the eluent could be found by increasing the adsorption–desorption cycles (Figure S3). These results revealed good regeneration capability. In addition, the FT-IR spectrum of PA-CS NFs after ten adsorption–desorption cycles showed little changes in comparison with origin nanofibers (Figure 7d), suggesting good stability.

### 3.8. Uranium Extraction from Natural Seawater

**Figure 8:**
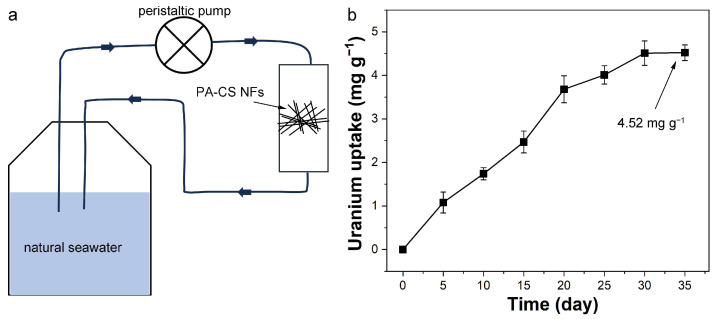
(**a**) Schematic uranium extraction device and (**b**) uranium extraction capacity of PA-CS NFs from natural seawater (uranyl ion concentration of 3.3 μg L^−1^, pH of 8.1).

Based on the above adsorption results, we further evaluated the uranium extraction ability of PA-CS NFs from natural seawater. The ion composition of the used natural seawater is listed in Table S2. A lab-made device was used to force natural seawater through the PA-CS NFs continuously (Figure 8a). A 35-day adsorption experiment was conducted, with measurements taken on day 5, 10, 15, 20, 25, 30, and 35. The uranium concentration in seawater exhibited a decreasing trend as the extraction time increased (Figure S4). Accordingly, the uranium extraction capacity of PA-CS NFs gradually increased over time and reached the saturation within 30 days. The final uranium extraction capacity of PA-CS NFs from natural seawater was 4.52 mg g^−1^ (Figure 8b), which was higher than many reported biomass-based adsorbents (Table 4). The results suggested that PA-CS NFs had the potential to extract uranium from real seawater.

### 3.9. Adsorption Mechanism Investigation

**Figure 9:**
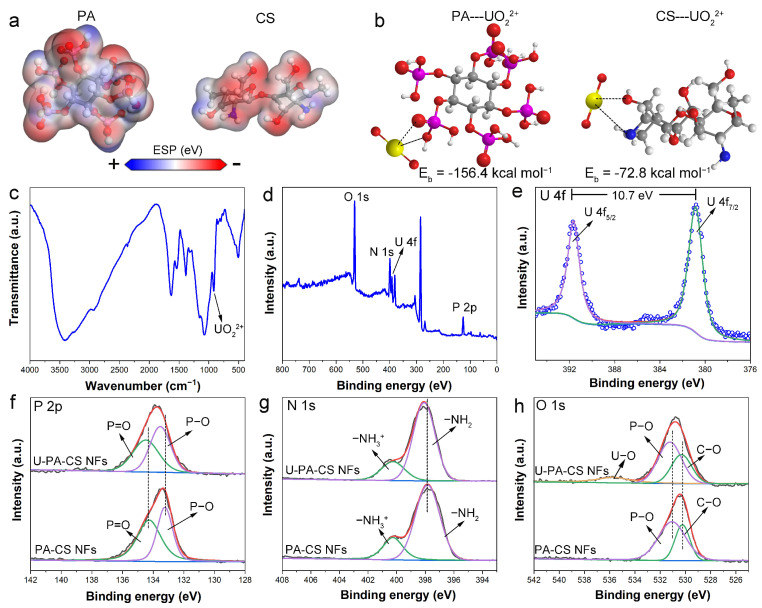
(**a**) ESP distribution of CS and PA fragments. (**b**) Structure optimization of adsorption model between UO_2_^2+^ and PA or CS fragment. (**c**) FT-IR spectrum, (**d**) XPS survey spectrum, and (**e**) high-resolution U 4f spectra of PA-CS NFs after uranium adsorption (U-PA-CS NFs). (**f**) High-resolution P 2p spectra, (**g**) high-resolution N 1s spectra, and (**h**) high-resolution O 1s spectra of PA-CS NFs before and after uranium adsorption.

To elucidate the adsorption mechanism, density functional theory (DFT) calculations were conducted. The model fragment for PA was its complete molecule and the model fragment for CS was the two repetitive fragments of its structural unit. The surface charge distributions of PA molecule and CS fragment were evaluated through electrostatic potential (ESP) analysis. As illustrated in Figure 9a, the oxygen sites in PA and both the oxygen and nitrogen sites in CS are negatively charged, indicating electron-rich properties. These electron-rich sites possessed strong chelation abilities that were considered as potential uranium adsorption sites [2]. Based on the electron-density surface, the optimal binding mode and binding energy (E_b_) between UO_2_^2+^ and PA molecule or CS fragment were calculated (Figure 9b). The absolute values of the binding energy followed the order of PA---UO_2_^2+^ > CS---UO_2_^2+^, which was consistent with the adsorption capacity trend of CS NFs < PA-CS NFs (Figure 5a). These results indicated that modification by PA could obviously improve the uranium uptake of chitosan and phosphate groups played a major role in binding uranyl ions by PA-CS NFs.

As shown in Figure 9c, the FT-IR analysis of PA-CS NFs after uranium adsorption (U-PA-CS NFs) revealed a strong peak at 921 cm^−1^, which is attributed to the characteristic of O=U=O [30]. This observation confirmed the adsorption of uranium onto PA-CS NFs. XPS analysis was also employed to investigate the binding mechanism. After uranium adsorption, characteristic U 4f peaks were observed in the XPS spectrum of U-PA-CS NFs (Figure 9d), agreeing with the FT-IR results. Furthermore, the U 4f peaks could be deconvoluted into two components corresponding to U 4f_5/2_ (391.6 eV) and U 4f_7/2_ (380.9 eV), respectively (Figure 9e), which were the typical peaks of hexavalent uranium [37]. These results indicated that the adsorption of uranyl ions was predominantly governed by coordination interactions rather than the redox reaction. Further fitting and analysis were performed on the high-resolution P 2p, N 1s, and O 1s spectra. The high-resolution P 2p spectra of both PA-CS NFs and U-PA-CS NFs could be deconvoluted into two peaks, attributed to P–O and P=O, respectively (Figure 9f). Similarly, the high-resolution N 1s spectra could be resolved into two peaks corresponding to –NH_2_ and –NH_3_^+^ (Figure 9g). The O 1s spectrum of PA-CS NFs exhibited two peaks assigned to P–O and C–O, respectively (Figure 9h). A new characteristic peak corresponding to U–O appeared in the spectra of U-PA-CS NFs in addition to the original oxygen environments. Moreover, after uranium adsorption, the binding energies of P–O, P=O, –NH_2_, and C–O all shifted to higher values. This increase in binding energy was due to electron transfer from these functional groups to U during the adsorption process [55]. The XPS results demonstrated that P–OH, P=O in PA and –NH_2_, and –OH in CS all participated in the coordination with uranyl ions. Based on the adsorption capacity comparison and DFT calculations, phosphate groups in PA served as the primary contributors to the PA-CS NFs’ adsorption capacity.

## 4. Conclusions

In summary, this work proposed a low-cost biomass adsorbent based on phytic acid modified chitosan nanofibers (PA-CS NFs), which were used to adsorb uranyl ions. Benefiting from the multiple functional groups (phosphate group, amino group, and hydroxyl group) and high porosity of electrospun nanofibers, PA-CS NFs exhibited fast adsorption rate, high adsorption capacity, satisfying selectivity, deep removal ability, and good recyclability. Importantly, PA-CS NFs with macroscopic membrane form demonstrated good practical utility, achieving the uranium extraction capacity of 4.52 mg g^−1^ from real seawater over 35 days. This performance was better than most of reported biomass adsorbents. Combining experimental characterizations and theoretical calculations, we concluded that though the phosphate group, amino group, and hydroxyl group all were involved in the uranyl binding, the phosphate group played the critical role. This work provides a novel insight into the development of biomass adsorbents with both economical and efficient uranium extraction from seawater.

## Figures and Tables

**Table 1:** Economic cost for fabrication of 1 kg of PA-CS NFs using industrial raw materials.

Raw Material	Price per Unit	Quantity (kg)	Total Price
chitosan	$14.3/kg	0.60 kg	$8.58
polyethylene oxide	$5.10/kg	0.17 kg	$0.87
phytic acid	$3.80/L	0.80 L	$3.04
acetic acid	$0.21/L	13.50 L	$2.84
other consumption	-- ^a^	--	$1.07
(labor, electricity, etc.)			
Total			$16.40

^a^ Not cover this part of the content.

**Table 2:** Langmuir and Freundlich constants of uranium adsorption by PA-CS NFs.

Isotherm Model	PA-CS NFs
Langmuir isotherm	
q_max_ (mg g^−1^)	457.8
b (L mg^−1^)	0.112
R^2^	0.9897
Freundlich isotherm	
K_F_	53.2
n	2.26
R^2^	0.8786

**Table 3:** Comparison of uranium adsorption performance of PA-CS NFs with other biomass adsorbents.

Adsorbents	Equilibrium Time (min) ^a^	C_0_ (mg L^−1^) ^b^	m/V (g L^−1^) ^c^	Adsorption Capacity (mg g^−1^) ^d^	C_0_ (mg L^−1^) ^e^	m/V (g L^−1^)	Ref., Year
tannic acid–chitosan hydrothermal carbon	250	25	0.4	96.7	10~100	0.4	[42], 2020
tannic acid/graphene oxide	120	167	0.6	266.6	10~170	0.6	[43], 2020
fungal mycelium microspheres	300	40~160	-- ^f^	250.7	25~175	--	[44], 2019
graphene oxide–chitosan composite	70	10	1.0	50.5	5~100	1.0	[45], 2017
forcespun chitosan nanofibers	180	086	0.5	110.0	10~130	0.5	[46], 2019
magnetic chitosan/graphene oxide	90	100	0.1	204.1	5~120	0.1	[47], 2018
functionalized attapulgite/chitosan aerogel	120	50	0.4	175.1	25~80	0.4	[48], 2018
chitosan modified phosphate rock	300	10	2.0	8.1	1~40	2.0	[49], 2018
phytic acid modified chitosan nanofibers	60	50	0.3	457.8	5~250	0.1	This work

^a^ The equilibrium time is obtained from the experimental kinetic data. ^b^ C_0_ is initial U concentration in the kinetic experiments. ^c^ m/v is the adsorbent mass to solution volume ratio. ^d^ Adsorption capacity is the theoretical uptake from the Langmuir isotherm model. ^e^ C_0_ is initial U concentration range in the isotherm experiments. ^f^ Not reported.

**Table 4:** Comparison of uranium extraction capacity of PA-CS NFs with other biomass adsorbents.

Adsorbents	Feed Solution (U Concentration)	Extraction Days	Extraction Capacity (mg g^−1^)	Ref., Year
chitosan functionalized aerogel	natural seawater (3.3 μg L^−1^)	28	3.59	[50], 2024
amidoximated bacteria cellulose	natural seawater (3.3 μg L^−1^)	2	0.39	[51], 2025
tannin loaded melamine foam	natural seawater (3.2 μg L^−1^)	16	4.20	[19], 2025
sodium alginate-based sponge	natural seawater (3.3 μg L^−1^)	14	3.58	[52], 2024
collagen fiber	natural seawater (3.3 μg L^−1^)	15	1.04	[53], 2024
hyaluronic acid conjugated biomass	natural seawater (3.3 μg L^−1^)	30	2.12	[54], 2021
phytic acid modified chitosan nanofibers	natural seawater (3.3 μg L^−1^)	35	4.52	This work

## Data Availability

The original contributions presented in this study are included in the article/Supplementary Material. Further inquiries can be directed to the corresponding authors.

## Supplementary Materials

The following supporting information can be downloaded at: https://www.mdpi.com/article/10.3390/polym17202725/s1. Figure S1. Stress-strain curve of PA-CS NFs. Figure S2. Residual weight of PA-CS NFs in real seawater adjusting with different pH values. Figure S3. Enrichment of uranium in the eluent as increasing the adsorption-desorption cycles. Figure S4. Uranium concentration changes in the seawater during the extraction by PA-CS NFs. Table S1. Kinetic parameters for adsorption of uranium by PA-CS NFs. Table S2. Concentrations of conventional elements in the used seawater.

## Abbreviations

The following abbreviations are used in this manuscript:

CS NFs	Chitosan nanofibers
EDS	Energy-dispersive X-ray spectroscopy
EDTA	Ethylenediaminetetraacetic acid
EPA	Environmental Protection Agency
ESP	Electrostatic potential
FT-IR	Fourier-transform infrared
ICP-MS	Inductively coupled plasma mass spectrometer
K_d_	Distribution coefficient
PA	Phytic acid
PA-CS NFs	Biomass nanofiber adsorbents
PEO	Poly(ethylene oxide)
SEM	Scanning electron microscopy
TEM	Transmission electron microscopy
TGA	Thermogravimetric analysis
UES	Uranium extraction from seawater
UO_2_^2+^	Uranyl ion
U-PA-CS NFs	Biomass nanofibers after uranium adsorption
XPS	X-ray photoelectron spectroscopy
