# Supplementary material for: Low-Cost Biomass Nanofibers from Chitosan and Phytic Acid for Efficient Uranium Extraction
Source: Polymers (Basel). 2025 Oct 10;17(20):2725. doi: 10.3390/polym17202725 (PMC12567019; doi:10.3390/polym17202725)
Supplement: Supplementary file 1 [file polymers-17-02725-s001.zip › polymers-3869045-supplementary.pdf]

Supporting Information

Low-Cost Biomass Nanofibers from Chitosan and Phytic Acid for Efficient Uranium Extraction

Zixu Ren [1,†], Dongqi Geng [1,†], Dingyang Chen [1], Minsi Shi [1], Qing Bai [2,*] and Rui Zhao [1,*]

[1] Key Laboratory of Polyoxometalate and Reticular Material Chemistry of Ministry of Education, Faculty of Chemistry, Northeast Normal University, Changchun 130024, China;
[2] College of New Materials and New Energies, Shenzhen Technology University, Shenzhen 518118, China;
* Correspondence: baiqing@sztu.edu.cn (Q.B.); zhaor814@nenu.edu.cn (R.Z.)
† These authors contributed equally to this work.

Kinetic analysis process

The kinetic data was analyzed by widely-used kinetic models (the pseudo-first-order kinetic model and the pseudo-second-order kinetic model), whose linear equations are listed as follows:

$$\log(q_e - q_t) = \log q_e - \frac{k_1 t}{2.303} \quad \text{(S1)}$$

$$\frac{t}{q_t} = \frac{1}{k_2 q_e^2} + \frac{t}{q_e} \quad \text{(S2)}$$

where q_t and q_e (mg g^{-1}) are the adsorption capacity at time t and equilibrium time, respectively. k_1 (h^{-1}) and k_2 (g h^{-1} mg^{-1}) are the pseudo-first order model rate constant and the pseudo-second order model rate constant, respectively.

Moreover, the kinetic data was also studied with intraparticle diffusion model (Weber-Morris model) to understand the rate controlling steps. Its linear form is shown as follows:

$$q_t = k_d \cdot t^{0.5} + L \quad \text{(S3)}$$

where k_d is the intra-particle diffusion rate constant and L is the thickness of boundary layer.

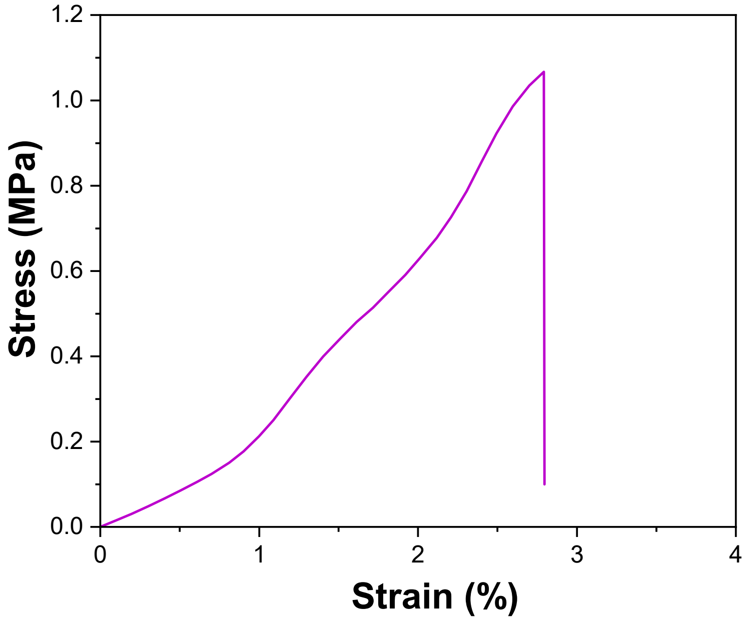

Figure S1. Stress-strain curve of PA-CS NFs.

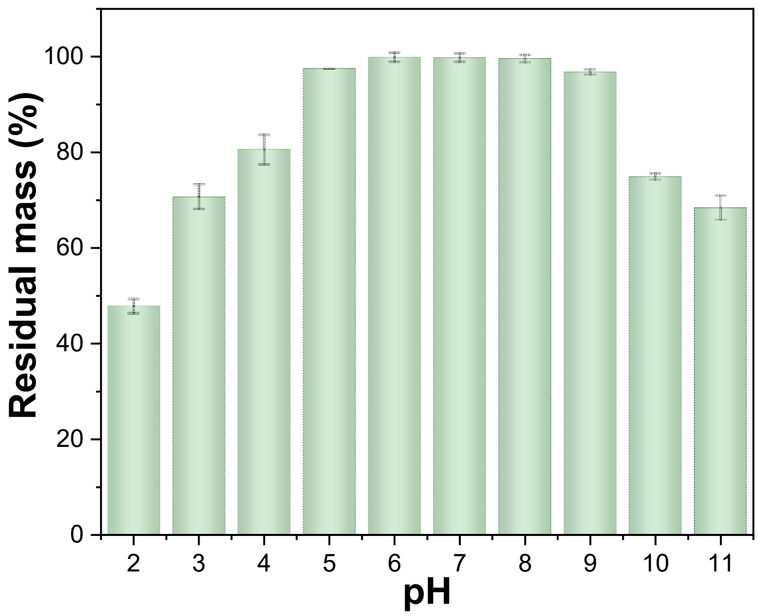

Figure S2. Residual weight of PA-CS NFs in real seawater adjusting with different pH values.

Table S1 Kinetic parameters for adsorption of uranium by PA-CS NFs.

Experimental	Pseudo-first-order model			Pseudo-second-order model		
q_{exp} (mg g^{-1})	q_e (mg g^{-1})	k_1 (min^{-1})	R^2	q_e (mg g^{-1})	k_2 (g mg^{-1} min^{-1})	R^2
152.2	100.8	0.055	0.9052	157.0	1.0×10^3	0.9999

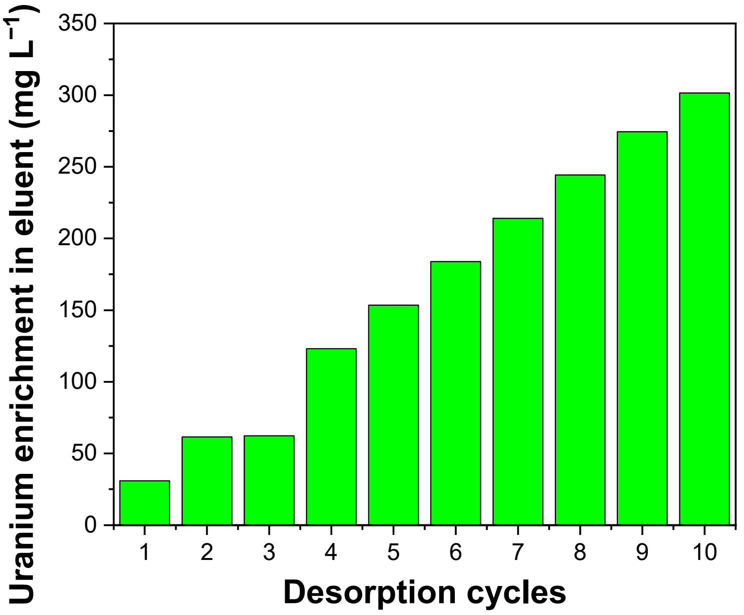

Figure S3. Enrichment of uranium in the eluent as increasing the adsorption-desorption cycles.

Table S2 Concentrations of conventional elements in the used seawater.

Element	Concentration (mg L^{-1})
Na	9787
Mg	1421
Ca	421
K	365
U	0.0033
V	0.0021
Fe	0.0035
Co	0.0006
Ni	0.0029
Cu	0.0006
Zn	0.0004

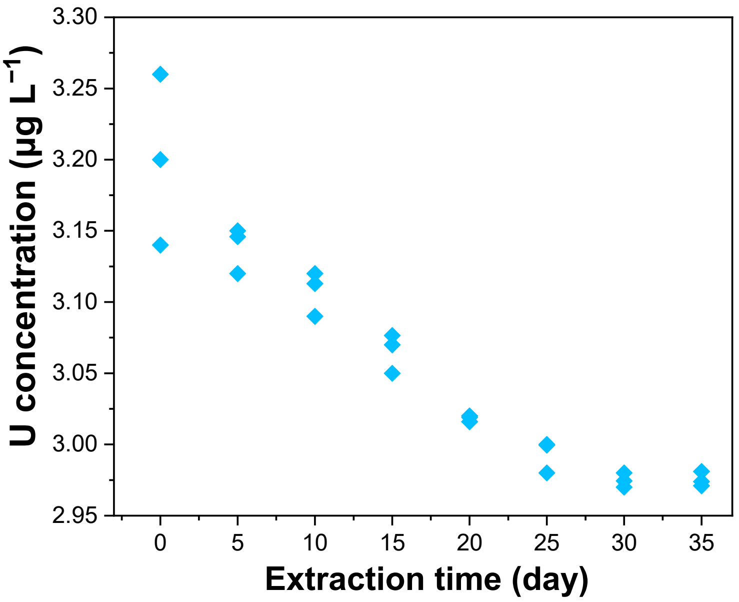

Figure S4. Uranium concentration changes in the seawater during the extraction by PA-CS NFs.
